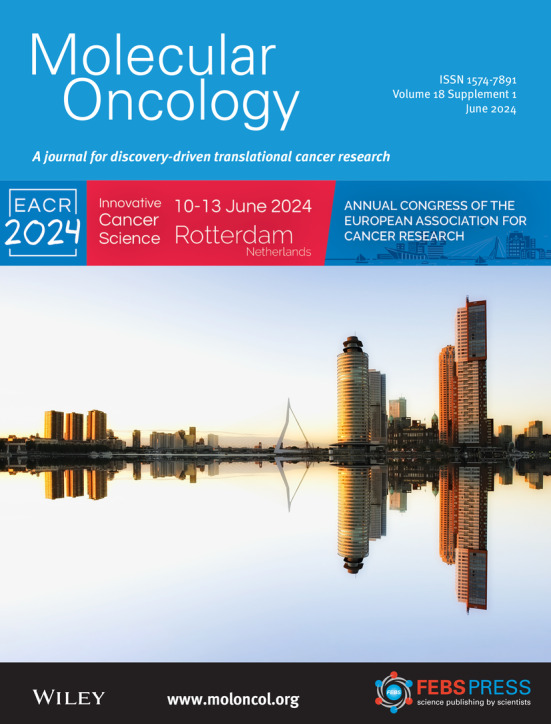# Issue Information

**DOI:** 10.1002/1878-0261.13682

**Published:** 2024-06-06

## Abstract

Abstracts submitted to the ‘EACR 2024 Congress: Innovative Cancer Science’, from 10‐13 June 2024 and accepted by the Congress Organising Committee are published in this Supplement of *Molecular Oncology*, an affiliated journal of the European Association for Cancer Research (EACR).